# Artificial light at night suppresses the metabolic response of a coral reef fish to a virtual predator

**DOI:** 10.1093/conphys/coag045

**Published:** 2026-07-27

**Authors:** Izzy C Tiddy, Manuel Vidal, Suzanne C Mills, Ricardo Beldade, Shaun S Killen

**Affiliations:** School of Biodiversity, One Health and Veterinary Medicine, College of Medical, Veterinary, and Life Sciences, University of Glasgow, University Avenue, Glasgow, G12 8QQ, Scotland; Institut de Neurosciences de la Timone, UMR 7289, Aix-Marseille Université, CNRS, 27, Boulevard Jean Moulin, 13005 Marseille, France; PSL Université Paris: EPHE-UPVD-CNRS, UAR 3278 CRIOBE, Papetoai, Mo’orea, BP 1013, 98729, French Polynesia; Laboratoire d’Excellence “CORAIL”, France; Institut Universitaire de France (IUF), France; Pontificia Universidad Católica de Chile, Facultad de Ciencias Biologicas, Avda. Libertador Bernardo O’Higgins, 340 Santiago, Chile; School of Biodiversity, One Health and Veterinary Medicine, College of Medical, Veterinary, and Life Sciences, University of Glasgow, University Avenue, Glasgow, G12 8QQ, Scotland

**Keywords:** Artificial light at night, coral reefs, metabolism, predation, virtual reality

## Abstract

Artificial light at night is an increasingly widespread feature of coastal environments and is known to alter animal physiology, but its effects on predator–prey interactions are poorly understood. Here, we show that chronic exposure to artificial light fundamentally alters metabolic responses to predation risk in a coral reef fish. Using immersive virtual reality to present a standardized visual predator stimulus while simultaneously measuring oxygen uptake, we quantified responses of wild-caught Polynesian anemonefish *Amphiprion maohiensis* with known histories of ambient or artificial nighttime light exposure. Fish from ambient light environments exhibited a strong anti-predator response, characterized by a suppression of metabolic rate (MR). In contrast, fish chronically exposed to artificial light increased their MR when faced with the same predator stimulus. This study demonstrates that artificial light can modify how physiological systems respond during ecologically critical interactions, making artificial light an important consideration when managing coastal systems. As light pollution expands globally, such disruption of predator–prey dynamics may have cascading consequences for energy budgets, survival and population persistence in coastal ecosystems.

## Abbreviations

ALAN: artificial light at night
MR: metabolic rate
VR: virtual reality

## Introduction

Anthropogenic pollutants are key stressors in many ecosystems. Non-chemical pollutants such as noise and artificial light at night (ALAN) may have significant physiological and fitness-related effects on animals (Mills *et al.*, 2020; Hillyer *et al.*, 2021; Schligler *et al.*, 2021, 2026a, 2026b; Tiddy *et al.*, 2024, 2026). These changes can have ecosystem-level effects due to altered relationships among and within species and between trophic levels (Davies *et al.*, 2014; Underwood *et al.*, 2017; Sanders *et al.*, 2020; Gaston and Sánchez de Miguel, 2022). As electrical lighting becomes a near-ubiquitous feature of human presence, the number of environments not exposed to some level of artificial light is decreasing rapidly (Sánchez de Miguel *et al.*, 2021). ALAN has been shown to have significant effects on physiology in animals such as fishes (Gaston and Sánchez de Miguel, 2022), including disruption of melatonin production, the hormone responsible for regulating circadian rhythms (Grubisic *et al.*, 2019; López-Olmeda *et al.*, 2019; Gaston and Sánchez de Miguel, 2022). This disruption has been linked to a rise in reactive oxygen species and cortisol levels in a reef fish species, blue-green chromis *Chromis viridis* exposed to 100 lx of ALAN (Zhao *et al.*, 2019; Hillyer *et al.*, 2021). Species including another reef fish *Dascyllus aruanus* (Georgiou *et al.*, 2024) become more active at night under ALAN (15–25 lx), and ALAN-induced (70 lx) changes in activity rhythms are associated with a rise in metabolic demand in a temperate intertidal fish, baunco *Girella laevifrons* (Pulgar *et al.*, 2019). ALAN exposure has been linked with changes to growth and survival (Schligler *et al.*, 2021, 2026a), as well as embryonic quality (Roost *et al.*, 2025) in anemonefish *Amphiprion maohiensis* (2–25 lx), and increased age at sexual maturity in Atlantic salmon *Salmo salar* (Leclercq *et al.*, 2011). While physiological effects of ALAN in fish species are relatively well documented, interactions between ALAN and responses to naturally occurring biotic stressors, such as predators, are less well-studied.

The ability to avoid predation is a key survival trait, and the physiological changes associated with different predator avoidance strategies vary widely. Active avoidance strategies may include fleeing (Geldart *et al.*, 2023), fast start escape responses (Witt *et al.*, 2015) or defence (Pitman *et al.*, 2001; Schligler *et al.*, 2022), and often occur alongside an increase in metabolic rate (MR) associated with tachycardia (increased heart rate) (Huuskonen and Karjalainen, 1997; Robison *et al.*, 2018). Other strategies, such as hiding and use of refugia, are often seen in animals such as reef fishes living in complex structures (Hixon and Beets, 1993; Rogers *et al.*, 2014; Nunes *et al.*, 2019), and may result in lower metabolic and heart rates (bradycardia) associated with a reduction in physical activity (Vallin *et al.*, 2006; Paul *et al.*, 2018). For example, lobsters reduced their routine metabolism by 31.4% when exposed to predation risk, suggesting an immobile anti-predator mechanism (Briceño *et al.*, 2018). In contrast, fathead minnows *Pimephales promelas* exposed to a conspecific alarm cue increased MR by 200%, consistent with preparation for an escape (Robison *et al.*, 2018).

If exposure to ALAN affects energy balance and metabolic demand, it may be expected that any changes in MR associated with predator exposure could be altered by ALAN exposure. For example, an increase in baseline metabolic costs may mean ALAN-exposed fish have a higher threshold for exhibiting a predator avoidance response, leading to less pronounced metabolic changes (Brown *et al.*, 2005; Behrens *et al.*, 2020). A study of golden grey mullet *Liza aurata* found increased recovery time in fish with lower response latencies to a predator cue (Killen *et al.*, 2015), the costs of which may be high for fish with elevated metabolic needs. Alternatively, increased stress following ALAN exposure may interact additively or synergistically with predation stress, leading to an amplified metabolic response (Gunderson *et al.*, 2016). Level of ALAN exposure (i.e., brightness) has been found to affect physiological and behavioural responses across taxa, particularly at low light levels (Schittko *et al.*, 2026), and may therefore modulate interactions with other ecological stressors. The impact of ALAN may also depend on the type of anti-predator response displayed, which is linked to the ecology of both the predator and prey species (Yoshida, 2021). Empirically testing these interactions is challenging, however, because delivering consistent and realistic predator stimuli in laboratory settings is difficult.

In this study, we used virtual reality (VR) simulation to assess the metabolic and behavioural responses of Polynesian anemonefish *A. maohiensis* to the risk of predation by Heller’s barracuda *Sphyraena helleri*. The use of VR has been recently demonstrated to elicit natural responses in a fish species, surgeonfish *Acanthurus triostegus* (Vidal *et al.*, 2023), and subsequently in damselfish and anemonefish (S.C.M. unpublished data) when exposed to predator and conspecific stimuli. VR is therefore a potentially effective technique to assess the effects of chronic stressors such as ALAN on responses to standardized stimuli in species whose predators are large and could not otherwise be used in lab-based trials (Vidal *et al.*, 2023). Precise control over predator appearance and behaviour also avoid confounding variation associated with live predators. Anemonefish rely partly on their anemones to provide predation protection (Mills *et al.*, 2020) and may respond to predators by hiding and reducing activity, leading to metabolic suppression (Paul *et al.*, 2018). Alternatively, anemonefish have also been found to actively attack predators (Schligler *et al.*, 2022), which may be associated with an increase in metabolic demand. Given emerging evidence that ALAN may affect both whole-organism and tissue-specific metabolism in fishes (Raoult *et al.*, 2012; Hillyer *et al.*, 2021), we hypothesized that ALAN exposure would modulate metabolic responses to predator cues.

## Materials and methods

### Ethical declarations

All fish collections were conducted under permits issued by the French Polynesian authorities (Arrêté n° 8286-MPR/DIREN and Arrêté n° 7445/MPR/DRM), within the framework of the BLEACHALAN and Raising Nemo projects. Ethical permits were granted by CNRS Animal Experimentation, R-13-CNRS-F1-16 to Yann Lacube and ANZCCART ComPass Animal Welfare Training certificate to S.C.M. Fish were transported from capture sites in small, perforated containers to allow water exchange and maintain oxygenation, and to minimize injury risk due to water movement within containers during transportation. Small containers were then placed in a thermally insulated cooler filled with a large volume of seawater to maintain water temperature throughout transportation. Tank size during holding at CRIOBE varied due to availability; however, all fish were kept in tanks with 1–3 others captured from the same site and were provided with an anemone within their tanks for enrichment. Tanks were constantly flushed with seawater and were cleaned daily to prevent build-up of waste.

### Study animals

In 2023, 2-month-old juvenile Polynesian anemonefish *A. maohiensis* (previously orangefin anemonefish *A. chrysopterus*) (O’Donnell *et al.*, 2025) were randomly placed in magnificent sea anemones, *Radianthus magnifica*, and distributed between three subtidal fringing reef locations around the island of Mo’orea, French Polynesia. The three locations were situated either on the north shore (Location 1: 17°29′01.9″S 149°50′37.8″W and Location 2: 17°28′47.6″S 149°48′13.8″W) or on the east shore of Mo’orea (Location 3: 17°30′05.3″S 149°45′51.1″W). Each location contained two sites exposed to one of two light treatments: ambient natural nighttime light or ALAN. Sites were situated 50–200 m apart ensuring similar conditions apart from ALAN exposure. The ALAN exposure sites were illuminated by spotlights above the water. Nocturnal light levels measured from 1800–0600 h under ambient conditions were consistently near-zero, and ranged from 4–21 lx on average at ALAN-exposed sites (Table 1). Full light data can be found in Tiddy *et al.* (2026).

**Table 1:** Mean light values from sampling locations under ambient light levels and ALAN, including number of overnight light samples taken at each site and number of fish collected

**Location**	**Treatment**	**Light level (mean ± SE), lx**	**Number of overnight samples**	**Number of fish**
1	ALAN	10.4 ± 0.14	8	5
	Ambient	0.006 ± 0.001	3	3
2	ALAN	4.27 ± 0.06	7	4
	Ambient	0.002 ± 0.0009	2	3
3	ALAN	21.2 ± 0.71	1	0
	Ambient	0.008 ± 0.002	2	2

In 2024, 17 Polynesian anemonefish were recaptured for this study (Supplementary Table S1). The majority of fish (*n* = 15) were recaptured from Locations 1 (*n* = 3 ambient, *n* = 5 ALAN) and 2 (*n* = 3 ambient, *n* = 4 ALAN) at 4–8 months old. Due to limited sample size for ambient fish meaning that sample sizes among light treatments were uneven (*n* = 6 ambient vs. *n* = 9 ALAN), an additional two fish from Location 3 (*n* = 2 ambient) were recaptured at 2 years of age. Size of study fish therefore varied significantly; however, this was accounted for in both the method of calculating MR and in statistical models (see Data processing and statistical analysis for details). Mean mass of fish in the ALAN treatment was 4.57 ± 0.81 g (mean ± SE), while the mean mass of fish in the ambient light treatment was higher at 9.02 ± 3.84 g due to the inclusion of two adult fish in this group (Supplementary Table S1). Following capture, fish were brought to CRIOBE station (17°31′08.1″S 149°50′58.9″W). Fish were kept in holding tanks for a minimum of three days (maximum holding time 12 days) prior to respirometry trials. As it was only possible to assay 2–3 fish per day, holding time co-varied closely with individual identity, which was included in models as a random effect. Fish from ambient anemones were held in ambient light conditions (12L:12D), whereas fish from ALAN-exposed anemones were continuously exposed to ALAN during the holding period, at a light level of 6.4 lx. All fish were fed in the morning and evening during holding but were fasted for 24 h prior to respirometry trials.

### Experimental setup

**Figure 1:**
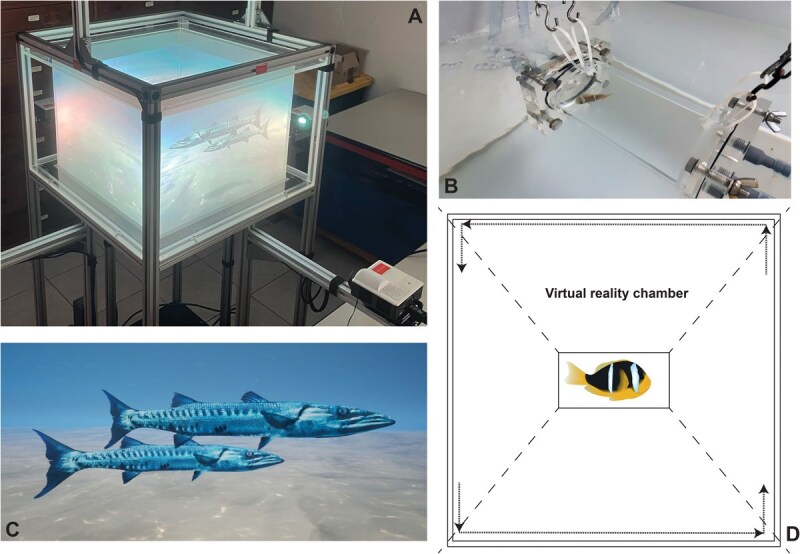
(A) Photograph showing VR chamber with sand and predator stimuli (sand only was shown during sand stimulus). (B) Photograph showing respirometry chamber containing *A. maohiensis*. (C) Image showing *S. helleri* (predator stimulus) as seen from within the VR chamber. (D) Schematic of VR chamber. Arrows indicate movement path of predator stimulus—stimulus side was randomized among trials

The experimental setup was an upgraded version of that described by Vidal *et al.* (2023). This consisted of a large tank (50 × 50 × 35 cm depth) made of 10 mm Plexiglas, the surfaces of which were coated with a translucent rear projection film to allow projection of VR images, but not allowing fish to see outside the tank (Fig. 1A). VR images were projected onto each side and the underside of the tank, using Optoma ML1050ST+ projectors running at 60 Hz with a resolution of 1280 × 800 (sides) and 800 × 800 (bottom). VR images were rendered using Unreal Engine 4.27 (EpicGames, 2022), which was also used to automate the timing and sequence in which images appeared. The tank was filled so that the top of the projected image aligned with the water surface (78 l total). We added realistic animated 3D models of adult predators, *S. helleri*, in our Unreal project. This model was bought on the internet and customized using Blender (version 3.2) free modelling software.

An acrylic respirometry chamber (Loligo Systems, Tiele, Denmark) with a volume of 917 ml was placed in the centre of the tank, suspended from a frame (Fig. 1B). This chamber was connected to a closed mixing circuit, which operated constantly (Supplementary Fig. S1). An oxygen probe within the mixing circuit connected to a FireSting oxygen meter, which was connected to a computer running Pyro Oxygen Logger (PyroScience, Aachen, Germany), allowing measurement of the oxygen level within the mixing circuit at intervals of 2 s (Supplementary Table S2). The chamber was also connected to a flush circuit, activated during the flush periods of the respirometry cycle only. This circuit exchanged water within the chamber with oxygenated water in the large VR tank. The closed phase of each respirometry cycle lasted 7 min, while the flush period lasted 3 min. Activation of the flush circuit was automated using Pyro Oxygen Logger. Water in the large VR tank was constantly passed through an ultraviolet filter to reduce bacterial growth within the setup. An Inkbird thermostat was used to regulate the temperature to 29°C (Supplementary Fig. S1). If temperatures fell below 29°C, a heating circuit began to operate, running water from the large VR tank through a heating coil to gradually warm the tank. The whole setup was treated daily with bleach, then rinsed with fresh water, prior to beginning trials, to reduce bacterial growth.

### Respirometry trials

MR was estimated in fish using intermittent flow respirometry (Killen *et al.*, 2021). Before fish were placed in the respirometry chamber, three blank cycles of the closed and flush phases were run to measure the level of background respiration in the setup. Fish were then individually placed in the respirometry chamber while a sandy environment was projected on the VR screens. Fish were allowed 60 min to acclimate to their surroundings, as time and logistical constraints prevented a longer acclimation. Fish were not provided with an anemone within the respirometry chamber due to the difficulty associated with tracking fish movement when a shelter is present. Oxygen uptake was then recorded for a further three cycles (30 min) with fish exposed to the static sandy environment. Each phase of the cycle was treated as one measurement of MR. Subsequently, fish were exposed to a predator stimulus consisting of two barracuda *S. helleri*, each indicating a total length of 115 cm with a maximum height of 30 cm, adjusted for distance to the focal fish. Barracuda were primarily projected on one side of the VR tank, parallel to the angle of the tank so the fish’s view of the predator was not obscured by tubing (Fig. 1C), but the predators were also projected on the adjoining screens during the approach and retreat phases. Predators appeared for 90 s at the start of each closed respirometry phase, consisting of a 20 s ‘approach’ (simulated travel distance 5 m), 50 s ‘static’ swimming parallel to the respirometry chamber and 20 s ‘retreat’ (simulated travel distance 5 m). This was repeated three times for each fish. Fish were recorded using a GoPro Hero Black 12 for 180 s at the start of each trial, i.e. during and immediately after predator appearance if applicable. Due to issues with video quality affecting fish tracking, however, six fish (*n* = 1 ambient; *n* = 5 ALAN) had to be excluded from activity analysis. This resulted in a low sample size and skewed size distribution across treatments; therefore, activity analysis was not included in the main findings of this study but can be found in the supplementary material. Fish were removed from the respirometry chamber immediately following the end of the third respirometry cycle with predator exposure. After the final fish of the day had undergone respirometry, a post-trial blank was run consisting of three cycles. Trials were run from 13 March 2024 to 22 March 2024. All trials were run during daylight hours.

### Data processing and statistical analysis

All analyses and data visualization were carried out in R version 3.5.2 (R Core Team, 2025). Data were processed using the FishResp, chron and dplyr packages (Morozov *et al.*, 2019; James and Hornik, 2023; Wickham *et al.*, 2023). FishResp was used to calculate absolute (mg O₂ h^−1^) and mass-specific (mg O₂ g^−1^ h^−1^) MR for each fish per closed cycle phase. Mass and volume of the fish and volume of the respirometry circuit were accounted for in MR calculations, assuming a fish density of 1 g cm^−3^ (Supplementary Table S2). MR values were adjusted according to the degree of background respiration found in the pre- and post-trial blank measurements (Supplementary Table S2). As fish mass varied, MR for each fish was adjusted to account for the nonlinear relationship between fish mass and mass-specific MR (Supplementary Table S2).

Statistical analysis was conducted using the glmmTMB package (Brooks *et al.*, 2017). Acclimation periods were removed from data prior to analysis, as inclusion of acclimation data would likely skew results due to stress associated with handling when the fish was first placed in the respirometry chamber. To investigate factors affecting MR, a linear mixed model was constructed with mass-adjusted MR (mg O₂ h^−1^) as the response variable. Explanatory variables were chronic light treatment (ambient/ALAN), VR projection (sand/predator) and cycle number (1, 2 or 3) per projection. Cycle number was included as a continuous variable to avoid unnecessarily increasing model degrees of freedom, and because we found no indication of non-linear effects of cycle number. The interactions between light treatment and VR projection and between VR projection and cycle number were also included. Model selection with and without the latter was carried out using the drop1() function in the stats package with a likelihood ratio test. Individual ID, nested within location from which individuals were collected, was included as a random effect variable. *Post hoc* analyses were carried out to examine relationships among levels of the interaction between light treatment and VR projection using the emmeans package (Lenth and Piaskowski, 2025) with a Tukey adjustment. Analyses were also re-run excluding the two adult fish from Location 3 to check that these did not bias results. As holding time in the lab varied, one additional model was constructed with mass-adjusted MR as the response variable and holding time in days as a continuous explanatory variable, to verify that MR was not affected by increasing holding time.

Model assumptions for all models were verified using the simulateResiduals() function in the DHARMa package (Hartig, 2016). As originally recommended by Fisher (1922), we use *P-*values as a continuous indicator of the strength of evidence for the alternate hypothesis and discuss results according to the language suggested in Muff *et al.* (2022).

## Results

**Figure 2:**
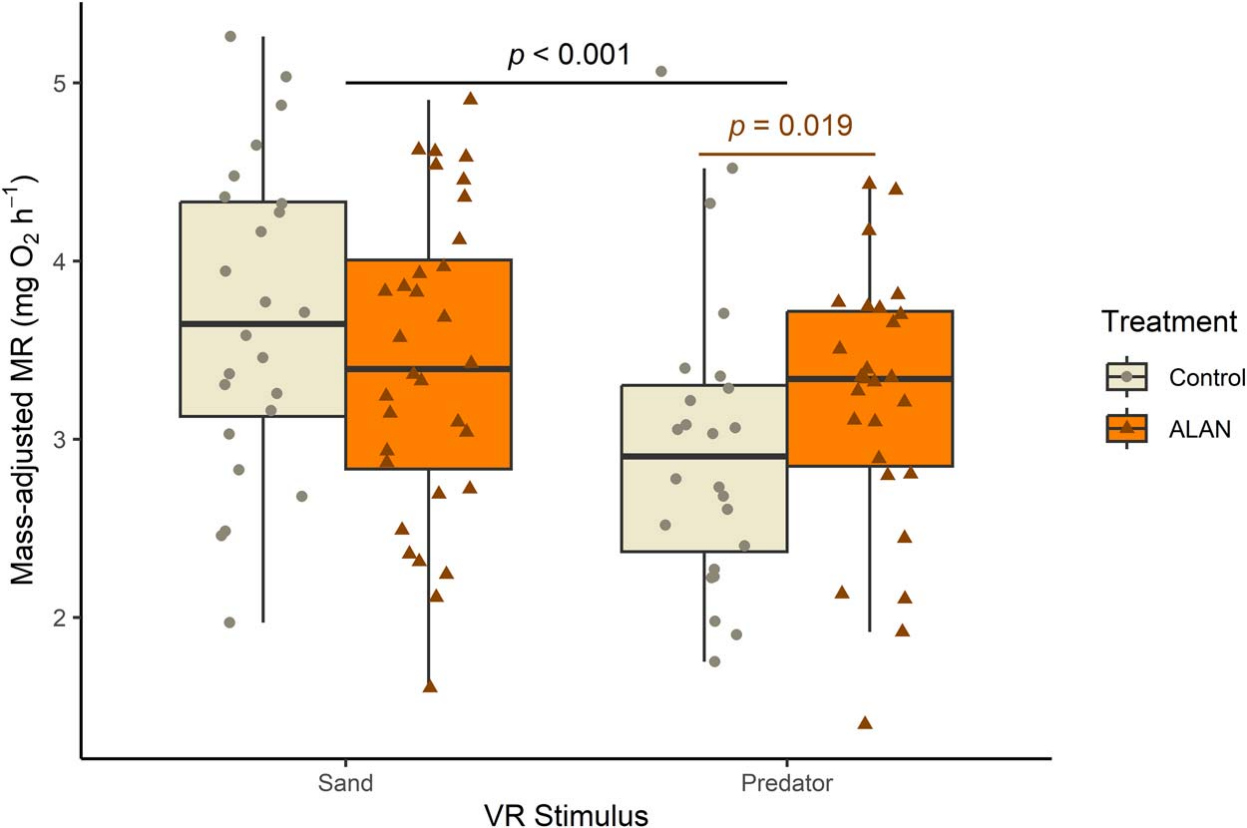
Effects of exposure to artificial light at night and exposure to a VR sandy environment or virtual predator on mass-adjusted MR

In fish exposed to ambient light, predator exposure produced a reduction in mass-adjusted MR compared with sand exposure (*z* = −5.225, *P* < 0.001; Table 2 and Fig. 2). While no overall effect of light treatment (ALAN vs ambient) was observed on MR during sand exposure, during predator exposure ALAN-exposed fish did not show a reduction in MR similarly to ambient light fish (*z* = 2.348, *P* = 0.019; Table 2 and Fig. 2; Supplementary Table S3). MR decreased during subsequent exposures to each VR projection (*z* = −3.444, *P* < 0.001; Table 2; Supplementary Fig. S2A). The interaction between VR projection and cycle number were not retained in the model. Excluding adult fish from the model reduced the strength of evidence for all trends (higher *P*-values; Supplementary Tables S4 and S5) but had no effect on the direction of trends. There was no effect of holding time on mass-adjusted MR (Supplementary Table S6). The results of preliminary activity analysis are shown in Supplementary Tables S7 and S8.

**Table 2:** Factors affecting mass-adjusted MR in *A. maohiensis* during and immediately after exposure to a virtual sandy environment or a virtual predator.

**Variable**	**Estimate**	**SE**	***z***	***P***	***R*** ^**2**^ _**m**_	***R*** ^**2**^ _**c**_
					0.130	0.691
Intercept	4.074	0.272	14.97	<0.001		
Light (ALAN)	−0.186	0.339	−0.549	0.583		
**VR (predators)**	**−0.719**	**0.138**	**−5.225**	<0.001^***^		
**Cycle number**	**−0.195**	**0.057**	**−3.444**	<0.001^***^		
**Light × VR (ALAN, predators)**	**0.438**	**0.186**	**2.348**	0.019^*^		

Significant effects at *p* < 0.05 are shown in bold. * indicates significance at *p* < 0.05; *** indicates significance at *p* < 0.001.

## Discussion

The use of VR is an exciting new technique in fish behaviour, allowing us to repeatedly study responses to standardized stimuli that would previously have been impossible to create in the laboratory (Vidal *et al.*, 2023). We found that *A. maohiensis* responded to a virtual predator through a reduction in mass-adjusted MR. This reduction in MR in response to a predator was not observed, however, in ALAN-exposed fish, indicating that anemonefish chronically exposed to ALAN may undergo an altered physiological response to a predator threat. Our study uses the novel opportunities afforded by VR technology to highlight the ecologically relevant effects of ALAN on fish physiology (Hillyer *et al.*, 2021; Schligler *et al.*, 2021; Gaston and Sánchez de Miguel, 2022).

While predator exposure may be expected to cause an increase in MR and other physiological parameters, such as heart rate associated with the flight escape response or defence (Huuskonen and Karjalainen, 1997), prey animals may also respond to perceived threats through metabolic suppression, sometimes associated with hiding or showing immobility behaviours (Paul *et al.*, 2018; Yoshida, 2021). This may serve other survival strategies, such as shelter use or to minimize detection prior to escaping (Vallin *et al.*, 2006; Paul *et al.*, 2018; Yoshida, 2021). In anemonefish, such as *A. maohiensis*, the use of anemones to hide from predators may mean that fish do not benefit from elevated MRs associated with preparing for a fast escape. While anemones were not provided to fish in respirometry chambers, our results indicate that a physiological response often associated with hiding persisted in fish raised under natural lighting. While anemones may play a key role in predator response in *A. maohiensis*, we did not include anemones in the setup as we planned to monitor activity, which would be challenging in fish that can hide in anemones. We cannot rule out that the absence of an anemone affected responses, however, and providing an anemone would be an important next step in determining the metabolic and associated behavioural response to predation stimuli in anemonefish.

Although *A. maohiensis* exposed to ambient light levels appeared to exhibit metabolic suppression in response to a predator stimulus, ALAN-treated individuals did not display a reduction in MR. Exposure to ALAN alters metabolic needs in fish (Pulgar *et al.*, 2019; Hillyer *et al.*, 2021); however, the mechanisms by which this translates to anti-predator responses are less clear. A study of metabolomic responses to ALAN exposure and predator presence in blue-green chromis *C. viridis* found that intermediates of energy-conversion pathways accumulated in the brains of ALAN-exposed fish, but this effect disappeared with predator exposure (Hillyer *et al.*, 2021). This was attributed to increased nocturnal activity in ALAN-exposed fish, which may have been reduced by the presence of a predator. However, ALAN-exposed fish also showed reduced levels of muscular glucose-producing animo acids, possibly indicating sustained swimming leading to protein degradation regardless of predator exposure (Moyes and West, 1995; Hillyer *et al.*, 2021). The metabolic effects of predation cues on ALAN-exposed fish may therefore vary across tissues and among biological levels, with the mechanisms of metabolic change likely relating to behavioural responses.

Although we were not able to fully assess behavioural changes associated with predator interactions in *A. maohiensis*, preliminary findings (Supplementary Fig. S3) indicate a suppressed behavioural response to predation cues in ALAN-exposed fish. Modelling MR with activity as an explanatory variable also revealed a possible change in the relationship between activity and MR in ALAN-exposed fish faced with a predator. If ALAN-exposed fish display an altered behavioural response during predator encounters in line with the altered metabolic response recorded here, these fish may be less likely to survive encounters if this relies on immobility or hiding (Schligler *et al.*, 2021, 2026a, 2026b). Increased metabolic needs resulting from maintaining a higher MR during predator encounters may also have secondary effects on behaviour and survivorship following encounters. Greater metabolic needs can be linked to increased activity and risk-taking behaviour associated with foraging (Metcalfe *et al.,* 2016). More time spent active and outside shelters may lead to increased predator encounters (Hulthén *et al.*, 2017), especially as both diurnal and nocturnal predatory species are more common at night under ALAN (Weschke *et al.*, 2024). While our study does not provide direct evidence of this, increased predator encounters in combination with reduced survival rates when encounters occur would have significant fitness effects on anemonefish in ALAN-exposed sites. More studies are urgently needed, therefore, to link physiological with behavioural and fitness outcomes.

Exposure to different predator cues may also elicit different metabolic responses. While we observed an immobility response to an ambush/sit-and-pursue predator, *S. helleri* (Porter and Motta, 2004; Grubich *et al.*, 2008; O’Toole *et al.*, 2010), fish may adopt a different strategy when encountering active predators. Aquatic insects undergo metabolic suppression in response to ambush but not active predators (Sommer *et al*., 2023). In fish, a model barracuda *Sphyraena barracuda* was found to inhibit herbivore foraging more than a sit-and-wait predator, black grouper *Mycteroperca bonaci*; however, metabolic or predator avoidance responses were not quantified (Catano *et al.*, 2017). A metanalysis by Preisser *et al.* (2007) found that sit-and-pursue predators caused greater impacts on life history traits such as growth and fecundity, indicating possible long-term physiological impacts. Future studies could make use of tools such as VR to expose fish to visual cues from different predator types, which may be associated with different metabolic and behavioural responses in prey species.

MR decreased with cycle number under both stimuli, i.e., with increasing time and increasing number of exposures to stimuli. This may result from relatively uniform habituation to sand and predation cues, as fish did not appear to habituate more rapidly to one cue or the other. Johnsson *et al.* (2001) found habituation to an aerial predator cue in Atlantic salmon *S. salar* after only two exposures, indicating that rapid habituation to predation cues can occur in fish. It is also possible, however that in the predator projections the increase in negative response may indicate an enhanced immobility response with subsequent predator exposures. While habituation might be expected after multiple exposures to a stimulus (Johnsson *et al.*, 2001; Goldenberg *et al.*, 2014; Nedelec *et al.*, 2016), three exposures may not be sufficient to induce habituation. Fish may therefore become more stressed and exhibit a more pronounced immobility response with subsequent predator exposures.

It would also be valuable to assess metabolic responses in adult *A. maohiensis*, as while two of our study animals were adults the remainder were juveniles. It is possible that adults may have differences in response that were not detected by our experiments, particularly as both adults came from the same location and were raised under the same light treatment. While mass was accounted for in calculations of MR and exclusion of adults from models did not affect trends, these adult individuals may have differed in other aspects of their response to predators and associated fitness implications (Schligler *et al.*, 2022). Future studies of the impacts of life stage on physiological and behavioural response to predation would be valuable both inherently and to allow studies to consider life stage of study organisms when designing protocols.

## Conclusions

Our findings demonstrate that ALAN may alter how coral reef fish respond physiologically to a predation threat. The change in response in MRs that we observed in ALAN-exposed fish during predator encounters could have cascading effects on energy budgets, foraging requirements and perhaps growth or reproductive investment, depending on the frequency at which fish encounter predator threats. By combining cutting-edge VR technology with physiological measurements, we have found evidence for subtle but important effects of anthropogenic stressors. As coastal development continues to increase globally, understanding interactions between artificial light pollution and predator–prey dynamics will be crucial for predicting and managing impacts on reef fish populations and their ecosystems. Future work should examine whether similar physiological impacts occur in other reef species and investigate the long-term consequences of this altered predator response for individual behaviour and fitness, and population dynamics.

## Supplementary Material

Web_Material_coag045

## Data Availability

Data are available at the Mendeley data repository: https://data.mendeley.com/datasets/hv8xj5yhgw/1.

## References

[ref1] Behrens JW, von Friesen LW, Brodin T, Ericsson P, Hirsch PE, Persson A, Sundelin A, van Deurs M, Nilsson PA (2020) Personality- and size-related metabolic performance in invasive round goby (*Neogobius melanostomus*). Physiol Behav 215: 112777. 10.1016/j.physbeh.2019.112777. 31857065

[ref2] Briceño F, Polymeropoulos E, Fitzgibbon Q, Dambacher J, Pecl G (2018) Changes in metabolic rate of spiny lobster under predation risk. Mar Ecol Prog Ser 598: 71–84. 10.3354/meps12644.

[ref3] Brooks ME, Kristensen K, van Benthem KJ, Magnusson A, Berg CW, Nielsen A, Skaug HJ, Mächler M, Bolker BM (2017) glmmTMB balances speed and flexibility among packages for zero-inflated generalized linear mixed modeling. R J 9: 378. 10.32614/RJ-2017-066.

[ref4] Brown C, Gardner C, Braithwaite VA (2005) Differential stress responses in fish from areas of high- and low-predation pressure. J Comp Physiol B 175: 305–312. 10.1007/s00360-005-0486-0. 15886993

[ref5] Catano LB, Barton MB, Boswell KM, Burkepile DE (2017) Predator identity and time of day interact to shape the risk–reward trade-off for herbivorous coral reef fishes. Oecologia 183: 763–773. 10.1007/s00442-016-3794-z. 28005174

[ref6] Davies TW, Duffy JP, Bennie J, Gaston KJ (2014) The nature, extent, and ecological implications of marine light pollution. Front Ecol Environ 12: 347–355. 10.1890/130281.

[ref7] EpicGames (2022) Unreal Engine (version 4.27). Available at: https://www.unrealengine.com/

[ref8] Fisher RA (1922) On the interpretation of χ^2^ from contingency tables, and the calculation of P. J R Stat Soc 85: 87. 10.2307/2340521.

[ref9] Gaston KJ, Sánchez de Miguel A (2022) Environmental impacts of artificial light at night. Annu Rev Env Resour 47: 373–398. 10.1146/annurev-environ-112420-014438.

[ref10] Geldart E, Love O, Gilchrist H, Barnas A, Harris C, Semeniuk C (2023) Heightened heart rate but similar flight responses to evolved versus recent predators in an Arctic seabird. Avian Conserv Ecol 18: art22. 10.5751/ACE-02445-180122.

[ref11] Georgiou D, Reeves SE, Burke da Silva K, Fobert EK (2024) Artificial light at night impacts night-time activity but not day-time behaviour in a diurnal coral reef fish. Basic Appl Ecol 74: 74–82. 10.1016/j.baae.2023.11.009.

[ref12] Goldenberg SU, Borcherding J, Heynen M (2014) Balancing the response to predation—the effects of shoal size, predation risk and habituation on behaviour of juvenile perch. Behav Ecol Sociobiol 68: 989–998. 10.1007/s00265-014-1711-1.

[ref13] Grubich JR, Rice AN, Westneat MW (2008) Functional morphology of bite mechanics in the great barracuda (*Sphyraena barracuda*). Zoology 111: 16–29. 10.1016/j.zool.2007.05.003. 18082386

[ref14] Grubisic M, Haim A, Bhusal P, Dominoni DM, Gabriel KMA, Jechow A, Kupprat F, Lerner A, Marchant P, Riley W et al. (2019) Light pollution, circadian photoreception, and melatonin in vertebrates. Sustainability 11: 6400. 10.3390/su11226400.

[ref15] Gunderson AR, Armstrong EJ, Stillman JH (2016) Multiple stressors in a changing world: the need for an improved perspective on physiological responses to the dynamic marine environment. Ann Rev Mar Sci 8: 357–378. 10.1146/annurev-marine-122414-033953. 26359817

[ref65] Hartig F (2016) DHARMa: Residual Diagnostics for Hierarchical (Multi-Level/Mixed). Regression Models. R Package (version 0.5.0). 10.32614/cran.package.dharma

[ref16] Hillyer KE, Beale DJ, Shima JS (2021) Artificial light at night interacts with predatory threat to alter reef fish metabolite profiles. Sci Total Environ 769: 144482. 10.1016/j.scitotenv.2020.144482. 33477042

[ref17] Hixon MA, Beets JP (1993) Predation, prey refuges, and the structure of coral-reef fish assemblages. Ecol Monogr 63: 77–101. 10.2307/2937124.

[ref18] Hulthén K, Chapman BB, Nilsson PA, Hansson L-A, Skov C, Brodersen J, Vinterstare J, Brönmark C (2017) A predation cost to bold fish in the wild. Sci Rep 7: 1239. 10.1038/s41598-017-01270-w. 28450699 PMC5430796

[ref19] Huuskonen H, Karjalainen J (1997) Predator-induced respiratory responses in juveniles of vendace *Coregonus albula*, whitefish *C. Lavaretus*, perch *Perca fluviatilis* and roach *Rutilus rutilus*. Environ Biol Fishes 49: 265–269. 10.1023/A:1007365332234.

[ref20] James D, Hornik K (2023) chron: chronological objects which can handle dates and times. R Package (version 2.3-62). 10.32614/CRAN.package.chron

[ref21] Johnsson JI, Höjesjö J, Fleming IA (2001) Behavioural and heart rate responses to predation risk in wild and domesticated Atlantic salmon. Can J Fish Aquat Sci 58: 788–794. 10.1139/f01-025.

[ref22] Killen SS, Christensen EAF, Cortese D, Závorka L, Norin T, Cotgrove L, Crespel A, Munson A, Nati JJH, Papatheodoulou M et al. (2021) Guidelines for reporting methods to estimate metabolic rates by aquatic intermittent-flow respirometry. J Exp Biol 224: jeb242522. 10.1242/jeb.242522. 34520540 PMC8467026

[ref23] Killen SS, Reid D, Marras S, Domenici P (2015) The interplay between aerobic metabolism and antipredator performance: vigilance is related to recovery rate after exercise. Front Physiol 6: 111. 10.3389/fphys.2015.00111. 25914648 PMC4391267

[ref24] Leclercq E, Taylor JF, Sprague M, Migaud H (2011) The potential of alternative lighting-systems to suppress pre-harvest sexual maturation of 1+ Atlantic salmon (*Salmo salar*) post-smolts reared in commercial sea-cages. Aquac Eng 44: 35–47. 10.1016/j.aquaeng.2010.12.001.

[ref25] Lenth RV, Piaskowski J (2025) emmeans: Estimated Marginal Means, aka Least-Squares Means. R Package (version 2.0.3). 10.32614/CRAN.package.emmeans

[ref26] López-Olmeda JF, Vera LM, Migaud H, López-Patiño MA, Míguez JM (2019) Environmental cycles, melatonin, and circadian control of stress response in fish. Front Endocrinol (Lausanne) 10: 279. 10.3389/fendo.2019.00279. 31244768 PMC6579845

[ref27] Metcalfe NB, Van Leeuwen TE, Killen SS (2016) Does individual variation in metabolic phenotype predict fish behaviour and performance? J Fish Biol 88: 298–321. 10.1111/jfb.12699. 26577442 PMC4991269

[ref28] Mills SC, Beldade R, Henry L, Laverty D, Nedelec SL, Simpson SD, Radford AN (2020) Hormonal and behavioural effects of motorboat noise on wild coral reef fish. Environ Pollut 262: 114250. 10.1016/j.envpol.2020.114250. 32443197

[ref29] Morozov S, McCairns RJS, Merilä J (2019) FishResp: R package and GUI application for analysis of aquatic respirometry data. Conserv Physiol 7: coz003. 10.1093/conphys/coz003. 30746152 PMC6364290

[ref30] Moyes CD, West TG (1995) Exercise metabolism of fish. In PW Hochachka, TP Mommsen, eds, Metabolic Biochemistry. Elsevier, Amsterdam, pp. 367–392

[ref31] Muff S, Nilsen EB, O’Hara RB, Nater CR (2022) Rewriting results sections in the language of evidence. Trends Ecol Evol 37: 203–210. 10.1016/j.tree.2021.10.009. 34799145

[ref32] Nedelec SL, Mills SC, Lecchini D, Nedelec B, Simpson SD, Radford AN (2016) Repeated exposure to noise increases tolerance in a coral reef fish. Environ Pollut 216: 428–436. 10.1016/j.envpol.2016.05.058. 27325546

[ref33] Nunes JACC, Leduc A, Miranda RJ, Cipresso PH, Alves JP, Mariano-Neto E, Sampaio CLS, Barros F (2019) Refuge choice specificity increases with predation risk in a rocky reef fish. J Exp Mar Biol Ecol 520: 151207. 10.1016/j.jembe.2019.151207.

[ref34] O’Donnell JL, Beldade R, Johns J, Bernardi G (2025) A new species of anemonefish from French Polynesia, *Amphiprion maohiensis*, (Pomacentridae, Amphiprioninae), the Polynesian anemonefish. Zookeys 1244: 225–237. 10.3897/zookeys.1244.141409. 40687518 PMC12272074

[ref35] O’Toole AC, Murchie KJ, Pullen C, Hanson KC, Suski CD, Danylchuk AJ, Cooke SJ (2010) Locomotory activity and depth distribution of adult great barracuda (*Sphyraena barracuda*) in Bahamian coastal habitats determined using acceleration and pressure biotelemetry transmitters. Mar Freshw Res 61: 1446. 10.1071/MF10046.

[ref36] Paul N, Novais SC, Lemos MFL, Kunzmann A (2018) Chemical predator signals induce metabolic suppression in rock goby (*Gobius paganellus*). PLoS One 13: e0209286. 10.1371/journal.pone.0209286. 30557310 PMC6296658

[ref37] Pitman RL, Ballance LT, Mesnick SI, Chivers SJ (2001) Killer whale predation on sperm whales: observations and implications. Mar Mamm Sci 17: 494–507. 10.1111/j.1748-7692.2001.tb01000.x.

[ref38] Porter HT, Motta PJ (2004) A comparison of strike and prey capture kinematics of three species of piscivorous fishes: Florida gar (*Lepisosteus platyrhincus*), redfin needlefish (*Strongylura notata*), and great barracuda (*Sphyraena barracuda*). Mar Biol 145: 989–1000. 10.1007/s00227-004-1380-0.

[ref39] Preisser EL, Orrock JL, Schmitz OJ (2007) Predator hunting mode and habitat domain alter nonconsumptive effects in predator–prey interactions. Ecology 88: 2744–2751. 10.1890/07-0260.1. 18051642

[ref40] Pulgar J, Zeballos D, Vargas J, Aldana M, Manriquez PH, Manriquez K, Quijón PA, Widdicombe S, Anguita C, Quintanilla D et al. (2019) Endogenous cycles, activity patterns and energy expenditure of an intertidal fish is modified by artificial light pollution at night (ALAN). Environ Pollut 244: 361–366. 10.1016/j.envpol.2018.10.063. 30352350

[ref41] Raoult V, Brown C, Zuberi A, Williamson JE (2012) Blood cortisol concentrations predict boldness in juvenile mulloway (*Argyosomus japonicus*). J Ethol 30: 225–232. 10.1007/s10164-011-0314-9.

[ref42] R Core Team (2025) R: A Language and Environment for Statistical Computing. *R Foundation for Statistical Computing*. Vienna, Austria. https://www.R-project.org/.

[ref43] Robison AL, Chapman T, Bidwell JR (2018) Predation cues influence metabolic rate and sensitivity to other chemical stressors in fathead minnows (*Pimephales promelas*) and *Daphnia pulex*. Ecotoxicology 27: 55–68. 10.1007/s10646-017-1870-8. 29101637

[ref44] Roelofs K, Dayan P (2022) Freezing revisited: coordinated autonomic and central optimization of threat coping. Nat Rev Neurosci 23: 568–580. 10.1038/s41583-022-00608-2. 35760906

[ref45] Rogers A, Blanchard JL, Mumby PJ (2014) Vulnerability of coral reef fisheries to a loss of structural complexity. Curr Biol 24: 1000–1005. 10.1016/j.cub.2014.03.026. 24746794

[ref46] Roost T, Hargous J, Van Espen L, Schligler J, Killen SS, Beldade R, Swearer SE, Mills SC (2025) Artificial light at night during early development directly affects embryonic but not larval quality in a wild coral reef fish. Conserv Physiol 13: coaf041. 10.1093/conphys/coaf041. 40584600 PMC12203906

[ref47] Sánchez de Miguel A, Bennie J, Rosenfeld E, Dzurjak S, Gaston KJ (2021) First estimation of global trends in nocturnal power emissions reveals acceleration of light pollution. Remote Sens 13: 3311. 10.3390/rs13163311.

[ref48] Sanders D, Frago E, Kehoe R, Patterson C, Gaston KJ (2020) A meta-analysis of biological impacts of artificial light at night. Nat Ecol Evol 5: 74–81. 10.1038/s41559-020-01322-x. 33139919

[ref49] Schittko C, Huggins B, Kiefer S, Kimmig S, Schroer S, Grubisic M, Hölker F (2026) Universal dose–response sensitivity of ecological processes to low levels of artificial light at night. Biol Conserv 318: 111825. 10.1016/j.biocon.2026.111825.

[ref50] Schligler J, Blandin A, Beldade R, Mills SC (2022) Aggression of an orange-fin anemonefish to a blacktip reef shark: a potential example of fish mobbing? Mar Biodivers 52: 17. 10.1007/s12526-022-01258-4.

[ref51] Schligler J, Cortese D, Beldade R, Swearer SE, Mills SC (2021) Long-term exposure to artificial light at night in the wild decreases survival and growth of a coral reef fish. Proc R Soc B: Biol Sci 288: 20210454. 10.1098/rspb.2021.0454. PMC8187998 34102892

[ref52] Schligler J, Norin T, McBride M, Morat F, Killen SS, Swearer SE, Beldade R, Mills SC (2026a) Fatal attraction: light pollution creates an ecological trap for wild fish. Glob Chang Biol 32: e70645. 10.1111/gcb.70645. 41486759

[ref53] Schligler J, Roost T, Schies J, McBride M, Swearer SE, Beldade R, Mills SC (2026b) Light pollution in the wild affects adult reef fish and has intergenerational and direct impacts on offspring. Proc R Soc B: Biol Sci 293: 20252225. 10.1098/rspb.2025.2225. 41633504

[ref54] Sommer NR, Alshwairikh YA, Arietta AZA, Skelly DK, Buchkowski RW (2023) Prey metabolic responses to predators depend on predator hunting mode and prey antipredator defenses. Oikos 2023: e09664. 10.1111/oik.09664.

[ref55] Tiddy IC, Cortese D, Munson A, Blewett TA, Killen SS (2024) Impacts of anthropogenic pollutants on social group cohesion and individual sociability in fish: a systematic review and meta-analysis. Environ Pollut 363: 125017. 10.1016/j.envpol.2024.125017. 39341410

[ref56] Tiddy IC, Killen SS, Mills SC (2026) Effects of artificial light at night on the diurnal risk-taking behaviour of a gregarious reef fish. Mar Ecol Prog Ser 786: 1–14. 10.3354/meps15137.

[ref57] Underwood CN, Davies TW, Queirós AM (2017) Artificial light at night alters trophic interactions of intertidal invertebrates. J Anim Ecol 86: 781–789. 10.1111/1365-2656.12670. 28452048

[ref58] Vallin A, Jakobsson S, Lind J, Wiklund C (2006) Crypsis versus intimidation—anti-predation defence in three closely related butterflies. Behav Ecol Sociobiol 59: 455–459. 10.1007/s00265-005-0069-9.

[ref59] Vidal M, Mills SC, Gairin E, Bertucci F, Lecchini D (2023) Validation of a novel immersive virtual reality set-up with responses of wild-caught freely moving coral reef fish. Anim Behav 206: 99–123. 10.1016/j.anbehav.2023.09.013.

[ref60] Weschke E, Schligler J, Hely I, Roost T, Schies J, Williams B, Dworzanski B, Mills SC, Beldade R, Simpson SD et al. (2024) Artificial light increases nighttime prevalence of predatory fishes, altering community composition on coral reefs. Glob Chang Biol 30: e70002. 10.1111/gcb.70002. 39692005 PMC11653166

[ref61] Wickham H, Francois R, Henry L, Muller K, Vaughan D (2023) dplyr: A grammar of data manipulation. R Package (version 1.2.1). 10.32614/CRAN.package.dplyr

[ref62] Witt WC, Wen L, Lauder GV (2015) Hydrodynamics of C-start escape responses of fish as studied with simple physical models. Integr Comp Biol 55: 728–739. 10.1093/icb/icv016. 25920507

[ref63] Yoshida M (2021) Immobility behaviours in fish: a comparison with other vertebrates. In M Sakai, ed, Death-Feigning in Insects: Mechanism and Function of Tonic Immobility. Springer, Singapore, pp. 159–178

[ref64] Zhao D, Yu Y, Shen Y, Liu Q, Zhao Z, Sharma R, Reiter RJ (2019) Melatonin synthesis and function: evolutionary history in animals and plants. Front Endocrinol (Lausanne) 10: 249. 10.3389/fendo.2019.00249. 31057485 PMC6481276

