# Supplementary material for: Artificial light at night suppresses the metabolic response of a coral reef fish to a virtual predator
Source: Conserv Physiol. 2026 Jul 27;14(1):coag045. doi: 10.1093/conphys/coag045 (PMC13402952; doi:10.1093/conphys/coag045)
Supplement: Web_Material_coag045 [file web_material_coag045.zip › Supplementary material.docx]

**Supplementary Table 1. Home location, treatment, age, body mass and ratio of body mass to respirometer volume of Polynesian anemonefish *Amphiprion maohiensis* used in respirometry trials. “Activity analysis” indicates whether fish were included in activity models, as some were excluded due to video tracking malfunctions.**

| Fish ID | Light treatment | Location | Age (months) | Body mass (g) | Body mass:  Respirometer volume | Activity analysis |
| --- | --- | --- | --- | --- | --- | --- |
| A630 | ALAN | 1 | 8 | 7.807 | 0.006 | No |
| A769 | ALAN | 1 | 6 | 5.427 | 0.005 | No |
| A408 | ALAN | 1 | 6 | 3.912 | 0.003 | No |
| C908 | Ambient | 1 | 6 | 2.451 | 0.002 | No |
| C923 | Ambient | 1 | 6 | 4.597 | 0.004 | Yes |
| C751 | Ambient | 1 | 8 | 3.590 | 0.003 | Yes |
| A11 | ALAN | 1 | 8 | 7.970 | 0.007 | No |
| A797 | ALAN | 1 | 8 | 6.566 | 0.005 | No |
| C917a | Ambient | 2 | 4 | 2.135 | 0.002 | Yes |
| C799b | Ambient | 2 | 8 | 4.072 | 0.003 | Yes |
| C924a | Ambient | 2 | 6 | 3.255 | 0.003 | Yes |
| A807b | ALAN | 2 | 4 | 2.550 | 0.002 | Yes |
| A310a | ALAN | 2 | 6 | 2.982 | 0.002 | Yes |
| A310c | ALAN | 2 | 4 | 2.031 | 0.002 | Yes |
| A914 | ALAN | 2 | 4 | 1.928 | 0.002 | Yes |
| C1 | Ambient | 3 | 48 | 30.98 | 0.026 | Yes |
| C2 | Ambient | 3 | 48 | 21.10 | 0.018 | Yes |

**Supplementary Table 2. Summary of key respirometry assay data, based on reporting guidelines found in Killen *et al*. (2021).**

| **Metric** | **Value(s)** |
| --- | --- |
| *Body mass of animals* | Body mass of all study animals is shown in Table 1 |
| *Volume of empty chamber* | 917 ml |
| *How chamber mixing was achieved* | Chamber mixing was achieved through a constantly running mixing circuit, shown in Supplementary Figure 1 |
| *Ratio of net respirometer volume to animal body mass* | Ratio of net respirometer volume to animal body mass is shown in table 1, assuming 1g of body mass per cm³ of fish |
| *Material of tubing in mixing circuit* | All tubing circuits were constructed from flexible PVC |
| *Volume of tubing in mixing circuit* | Tubing in the mixing circuit was 367 cm in length by 10 mm in diameter, giving a total volume of 288.44 ml |
| *Confirm volume of tubing used in O_2_ calculations* | Total volume used in calculations was the chamber volume added to the mixing circuit volume for a total volume of 1205.44 ml |
| *Material of respirometer* | The respirometry chamber consisted of transparent hard acrylic |
| *Type of O_2_ probe and data recording* | A Firesting oxygen probe was used, connected to a laptop running the OxyReg software as shown in Figure 1 |
| *Sampling frequency of DO₂* | DO₂ was sampled every 2 seconds |
| *Placement of O₂ probe* | The oxygen probe was placed in the mixing circuit as shown in Figure 1 |
| *Flow rate during flushing and recirc* | During closed phases the maximum flow rate was 2280 l h⁻¹; this was double during the flush period as two pumps were running |
| *Timing of flush/closed cycles* | Flush cycles were 3 minutes long; closed cycles were 7 minutes long |
| *Wait time excluded from closed cycles* | 30 seconds |
| *Frequency and method of probe calibration* | The oxygen probe was calibrated daily to 100% oxygen |
| *State whether software temperature compensation was used during recording of water oxygen concentration* | OxyReg software temperature compensation was used during oxygen recording |
| *Temperature during respirometry* | 29°C |
| *How temperature was controlled* | A temperature coil placed in a sump and connected to a thermostat was used to regulate temperature as shown in Figure 1 |
| *Photoperiod during respirometry* | 12L:12D |
| *Describe if (and how) the ambient water bath was cleaned and aerated during measurement of oxygen uptake* | The ambient water bath was constantly cleaned by passing water through a UV filter during all trials. The bath was aerated using an airstone placed alongside the respirometry chamber (Figure 1) |
| *Total volume of ambient water bath and any associated reservoirs* | The total volume of the water bath was 78 l |
| *Minimum O_2_ reached during closed phases* | The minimum O₂ level reached during closed phases was 4.8026 mg l⁻¹ |
| *Were chambers visually shielded from disturbance* | Walls of the VR chamber were coated with a translucent rear projection film, preventing the fish from seeing outside the VR chamber |
| *How many animals measured at once* | Animals were measured one-by-one |
| *Duration of fasting* | Fish were fasted for 24h prior to testing |
| *Duration of all trials combined* | 10 days |
| *Acclimation time to lab* | Minimum 5 days |
| *State whether background microbial respiration was measured and accounted for, and if so, method used* | Background respiration was measured before the first trial of the day, and after the last trial of the day. |
| *If background respiration was measured at beginning and/or end, state how many slopes and for what duration* | Background respiration was calculated from one closed phase slope before the first trial, and one closed phase slope after the final trial. |
| *State how changes in background respiration were modelled over time* | Changes were modelled linearly over time |
| *Provide level of background respiration* | Level of background respiration averaged -28% of fish respiration rate |
| *State method and frequency of system cleaning* | The entire setup was bleached daily prior to trials being carried out |
| *Acclimation period* | 60 mins |
| *Time period over which O_2_ was measured (trial total)* | 60 mins |
| *State value taken as SMR/RMR* | Baseline MR was taken as the rate of oxygen consumption during control treatments |
| *Total slopes measured and used* | 3 for control; 3 for predator |
| *State times removed from calculations* | Acclimation periods were removed from calculations |
| *R^2^ threshold for slopes used* | 0.9 |
| *Proportion of data removed due to being below R^2^ threshold* | One slope had to be removed from a total of 102 slopes i.e., <1% |
| *Sample size* | 17 |
| *How O_2_ uptake rates calculated* | mg O₂ g⁻¹ in FishResp |
| *Confirm vol/mass of animal subtracted when calculating O2 uptake* | The volume of the animal was subtracted when calculated oxygen uptake |
| *Specify whether variation in body mass was accounted for in analyses and describe any allometric body-mass correction or adjustment* | Metabolic rate was calculated as mass-adjusted MR. The log-log relationship between mass and absolute metabolic rate was modelled, and model residuals were combined with the predicted metabolic rate of a fish of mean mass to give an adjusted metabolic rate for each individual |

**Supplementary Table 3. Results of post-hoc emmeans test comparing levels of the interaction between VR projection and light treatment on mass-specific metabolic rate (MR) in *Amphiprion maohiensis* during and immediately after exposure to a sand or predator projection.**

| Variable | Estimate | Std. error | z ratio | *p* |
| --- | --- | --- | --- | --- |
| Ambient Sand –ALAN Sand | 0.186 | 0.339 | 0.549 | 0.947 |
| **Ambient Sand –**  **Ambient Predator** | **0.719** | **0.138** | **5.225** | **< 0.001***** |
| Ambient Sand –  ALAN Predator | 0.467 | 0.340 | 1.374 | 0.516 |
| ALAN Sand –  Ambient Predator | 0.533 | 0.339 | 1.575 | 0.393 |
| ALAN Sand –  ALAN Predator | 0.281 | 0.126 | 2.239 | 0.113 |
| Ambient Predator –  ALAN Predator | -0.252 | 0.340 | -0.740 | 0.881 |

**Supplementary Table 4. Factors affecting mass-adjusted metabolic rate (MR) in *Amphiprion maohiensis* juveniles only during and immediately after exposure to a virtual sandy environment or a virtual predator.**

| Variable | Estimate | Std. error | z | *p* | R^2^_m_ | R^2^_c_ |
| --- | --- | --- | --- | --- | --- | --- |
|  |  |  |  |  | 0.096 | 0.704 |
| Intercept | 2.821 | 0.229 | 12.31 | < 0.001 |  |  |
| Light (ALAN) | -0.116 | 0.274 | -0.424 | 0.672 |  |  |
| **VR (Predators)** | **-0.467** | **0.112** | **-4.150** | **< 0.001***** |  |  |
| **Cycle number** | **-0.120** | **0.043** | **-2.824** | **0.005**** |  |  |
| **Light*VR (ALAN, Predators)** | **0.268** | **0.143** | **1.868** | **0.062** |  |  |

**Supplementary Table 5. Results of post-hoc emmeans test comparing levels of the interaction between VR projection and light treatment on mass-specific metabolic rate (MR) in *Amphiprion maohiensis* juveniles only during and immediately after exposure to a sand or predator projection.**

| Variable | Estimate | Std. error | z ratio | *p* |
| --- | --- | --- | --- | --- |
| Ambient Sand –ALAN Sand | 0.116 | 0.274 | 0.424 | 0.974 |
| **Ambient Sand –**  **Ambient Predator** | **0.467** | **0.112** | **4.150** | **< 0.001***** |
| Ambient Sand –  ALAN Predator | 0.315 | 0.275 | 1.146 | 0.661 |
| ALAN Sand –  Ambient Predator | 0.351 | 0.274 | 1.280 | 0.575 |
| ALAN Sand –  ALAN Predator | 0.199 | 0.089 | 2.237 | 0.114 |
| Ambient Predator –  ALAN Predator | -0.152 | 0.275 | -0.552 | 0.946 |

**Supplementary Table 6. Results of model testing effects of holding time at CRIOBE on mass-adjusted metabolic rate in *Amphiprion maohiensis*.**

| Variable | Estimate | Std. error | z | *p* |
| --- | --- | --- | --- | --- |
| Intercept | 3.119 | 0.233 | 13.36 | < 0.001 |
| Holding time (days) | 0.027 | 0.028 | 0.964 | 0.335 |

**Supplementary Methods**

Videos were processed using EthoVision movement tracking software (Noldus et al., 2001). Distance moved (cm) and time spent moving (s) were analysed for each video. Time spent moving was then converted to a percentage of total trial time. Two models were constructed to investigate factors affecting activity level. The response variables for these models were distance moved (cm) and time spent moving (%) in the first 180 seconds of each closed respirometry phase. Distance moved was natural log transformed to fulfil model assumptions. Explanatory variables were chronic light treatment (ambient/ALAN), VR projection (sand/predator) and cycle number (1, 2, or 3) per projection. Cycle number was included as a continuous variable to avoid unnecessarily increasing model degrees of freedom, and because we found no indication of non-linear effects of cycle number. The interactions between light treatment and VR projection and between VR projection and cycle number were also included. Model selection with and without the latter was carried out using the drop1() function in the stats package with a likelihood ratio test. Individual ID, nested within location from which individuals were collected, was included as a random effect variable. Post-hoc analyses were carried out to examine relationships among levels of the interaction between light treatment and VR projection using the emmeans package (Lenth & Piaskowski, 2025) with a Tukey adjustment.

Two models were constructed to determine effects of treatment and VR projection on the relationship between activity level and metabolic rate. The response variable in each of these models was mass-adjusted MR. The explanatory variables were light treatment, VR projection, cycle number, and either distance moved or percentage time spent moving. The interaction between activity level (distance moved or time spent moving) and cycle number, and a three-way interaction between light treatment, VR projection, and activity level, were also included in full models. Individual ID nested within collection site ID was included as a random effect variable. Model selection with and without the interaction between activity level and cycle number was carried out using drop1(). Distance moved was rescaled for use as an explanatory variable.

**Supplementary Results**

The interactions between VR projection and mass or cycle number were not retained in either activity model. Both distance moved (z = -3.290, *p* < 0.001; Supplementary Table 7; Supplementary Fig. 3A) and time spent moving (z = -4.012, *p* < 0.001; Supplementary Table 7; Supplementary Fig. 3B) were reduced in predator compared with sand projections in fish exposed to ambient light. Chronic light treatment alone (i.e., ALAN exposure) had no effect on either distance moved or time spent moving; however, there was weak evidence that ALAN-exposed fish spent more time moving when faced with a predator (z = 1.897, *p* = 0.058; Supplementary Table 7; Supplementary Fig. 3B). There was moderate evidence that distance moved decreased with subsequent exposures to VR projections (z = -2.040, *p* = 0.042; Supplementary Table 7; Supplementary Fig. 2B), but no evidence that time spent moving decreased.

Both distance moved (z = 4.611, *p* < 0.001; Supplementary Table 8; Supplementary Fig. 3C-D) and percentage time moving (z = 4.947, *p* < 0.001) were positively correlated with mass-adjusted MR. There was moderate evidence that the positive relationship between distance moved and MR was weaker in ALAN-exposed fish during to the predator stimulus (z = -2.440, *p* = 0.015; Supplementary Table 8; Supplementary Fig. 3C-D), and weak evidence for a similar effect in the time moving model (z = 1.836, *p* = 0.059). Metabolic rate was reduced during predator projections in fish exposed to ambient light (distance moved model; Supplementary Table 8). There was also weak evidence for an increase in MR in ALAN-exposed fish during the predator stimulus (time moving model; Supplementary Table 8). Cycle number was found to be negatively correlated with MR in both models (Supplementary Table 8). The interaction between activity level and cycle number was not retained in either model.

**Supplementary Table 7. Factors affecting activity metrics in *Amphiprion maohiensis* during and immediately after exposure to a virtual sandy environment or a virtual predator.**

| Response variable | Variable | Estimate | Std. error | z | *p* | R^2^_m_ | R^2^_c_ |
| --- | --- | --- | --- | --- | --- | --- | --- |
|  |  |  |  |  |  | 0.156 | 0.619 |
| *Distance moved* | Intercept | 6.835 | 0.157 | 43.53 | < 0.001 |  |  |
|  | Light (ALAN) | -0.124 | 0.189 | -0.660 | 0.512 |  |  |
|  | **VR (Predators)** | **-0.228** | **0.069** | **-3.290** | **< 0.001***** |  |  |
|  | Mass | -0.011 | 0.009 | -1.130 | 0.258 |  |  |
|  | **Cycle number** | **-0.069** | **0.034** | **-2.040** | **0.042*** |  |  |
|  | Light*VR (ALAN, Predators) | 0.171 | 0.115 | 1.490 | 0.136 |  |  |
|  |  |  |  |  |  | 0.133 | 0.573 |
| *Time spent moving (%)* | Intercept | 70.35 | 6.813 | 10.33 | < 0.001 |  |  |
|  | Light (ALAN) | -1.932 | 8.129 | -0.238 | 0.812 |  |  |
|  | **VR (Predators)** | **-12.74** | **3.176** | **-4.012** | **< 0.001***** |  |  |
|  | Mass | 0.032 | 0.404 | 0.079 | 0.937 |  |  |
|  | Cycle number | -2.437 | 1.552 | -1.570 | 0.116 |  |  |
|  | **Light*VR (ALAN, Predators)** | **9.994** | **5.267** | **1.897** | **0.058** |  |  |
|  |  |  |  |  |  |  |  |

**Supplementary Table 8. Models linking mass-adjusted metabolic rate (MR) with activity level in *Amphiprion maohiensis* during and immediately after exposure to a virtual sandy environment or a virtual predator.**

| *Response variable* | Variable | Estimate | Std. error | z | *p* | R^2^_m_ | R^2^_c_ |
| --- | --- | --- | --- | --- | --- | --- | --- |
|  |  |  |  |  |  | 0.611 | 0.870 |
| *MR* | Intercept | 4.174 | 0.211 | 19.81 | < 0.001 |  |  |
|  | Light (ALAN) | -0.307 | 0.301 | -1.018 | 0.309 |  |  |
|  | **VR (Predators)** | **-0.530** | **0.106** | **-5.000** | **< 0.001***** |  |  |
|  | **Cycle number** | **-0.204** | **0.050** | **-4.082** | **< 0.001***** |  |  |
|  | **Distance moved** | **0.490** | **0.106** | **4.611** | **< 0.001***** |  |  |
|  | Light (ALAN)  *Distance | 0.309 | 0.214 | 1.441 | 0.150 |  |  |
|  | VR (Predator)*  Distance | 0.074 | 0.115 | 0.640 | 0.522 |  |  |
|  | Light (ALAN)*  VR (Predator) | 0.092 | 0.167 | 0.549 | 0.583 |  |  |
|  | **Light (ALAN)***  **VR (Predator)***  **Distance** | **-0.450** | **0.184** | **-2.440** | **0.015*** |  |  |
|  |  |  |  |  |  | 0.568 | 0.871 |
| *MR* | Intercept | 2.205 | 0.485 | 4.547 | < 0.001 |  |  |
|  | Light (ALAN) | -0.776 | 0.811 | -0.956 | 0.339 |  |  |
|  | VR (Predators) | -0.610 | 0.453 | -1.347 | 0.178 |  |  |
|  | **Cycle number** | **-0.198** | **0.047** | **-4.174** | **< 0.001***** |  |  |
|  | **Percent moving** | **0.031** | **0.006** | **4.947** | **< 0.001***** |  |  |
|  | Light (ALAN)  *Percent moving | 0.008 | 0.012 | 0.678 | 0.498 |  |  |
|  | VR (Predator)*  Percent moving | 0.004 | 0.007 | 0.530 | 0.596 |  |  |
|  | **Light (ALAN)***  **VR (Predator)** | **1.322** | **0.720** | **1.836** | **0.066** |  |  |
|  | **Light (ALAN)***  **VR (Predator)***  **Percent moving** | **-0.021** | **0.011** | **-1.886** | **0.059** |  |  |
|  |  |  |  |  |  |  |  |


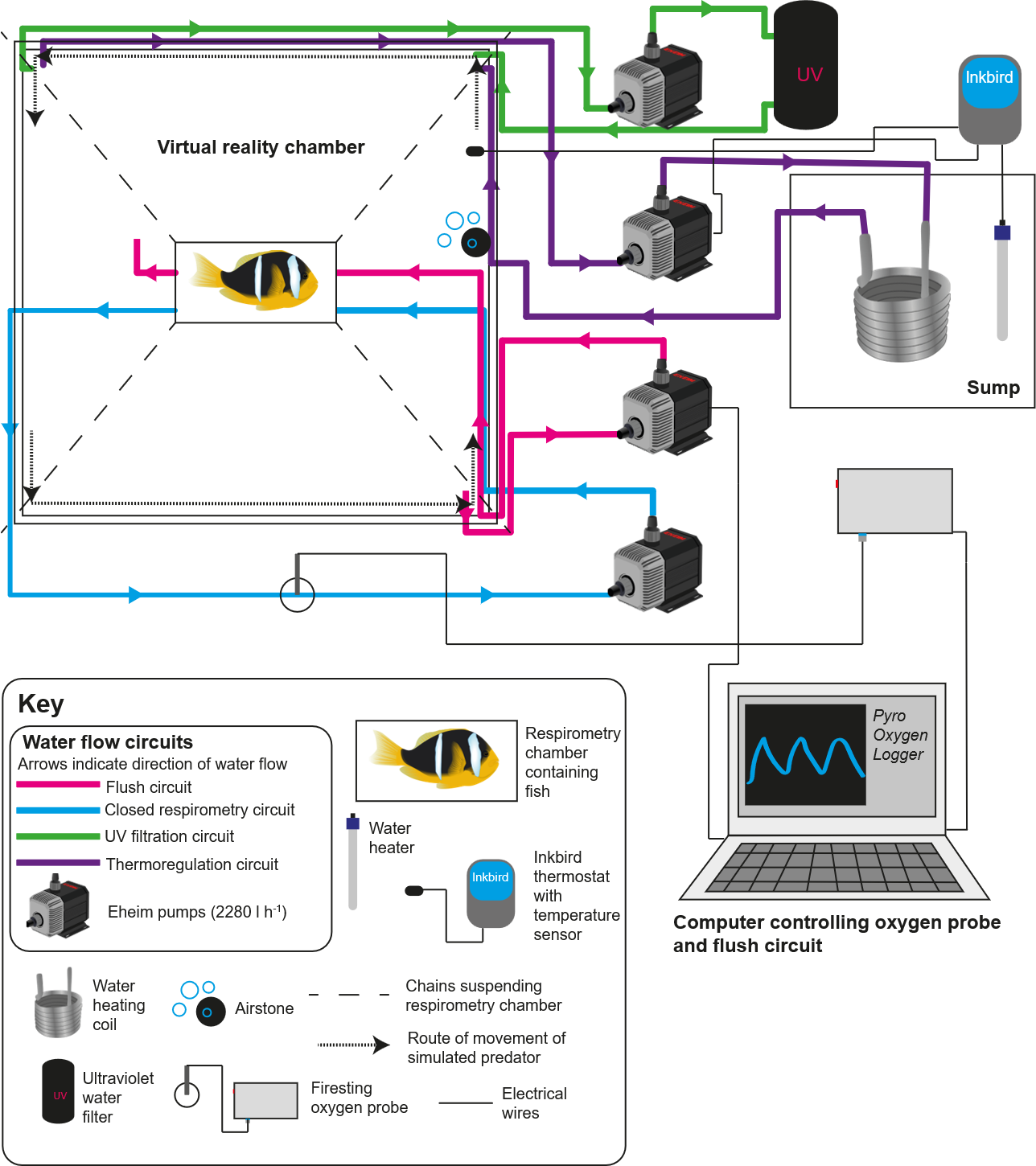


**Supplementary Fig. 1. Diagram showing setup of respirometry chamber within virtual reality simulation chamber.**


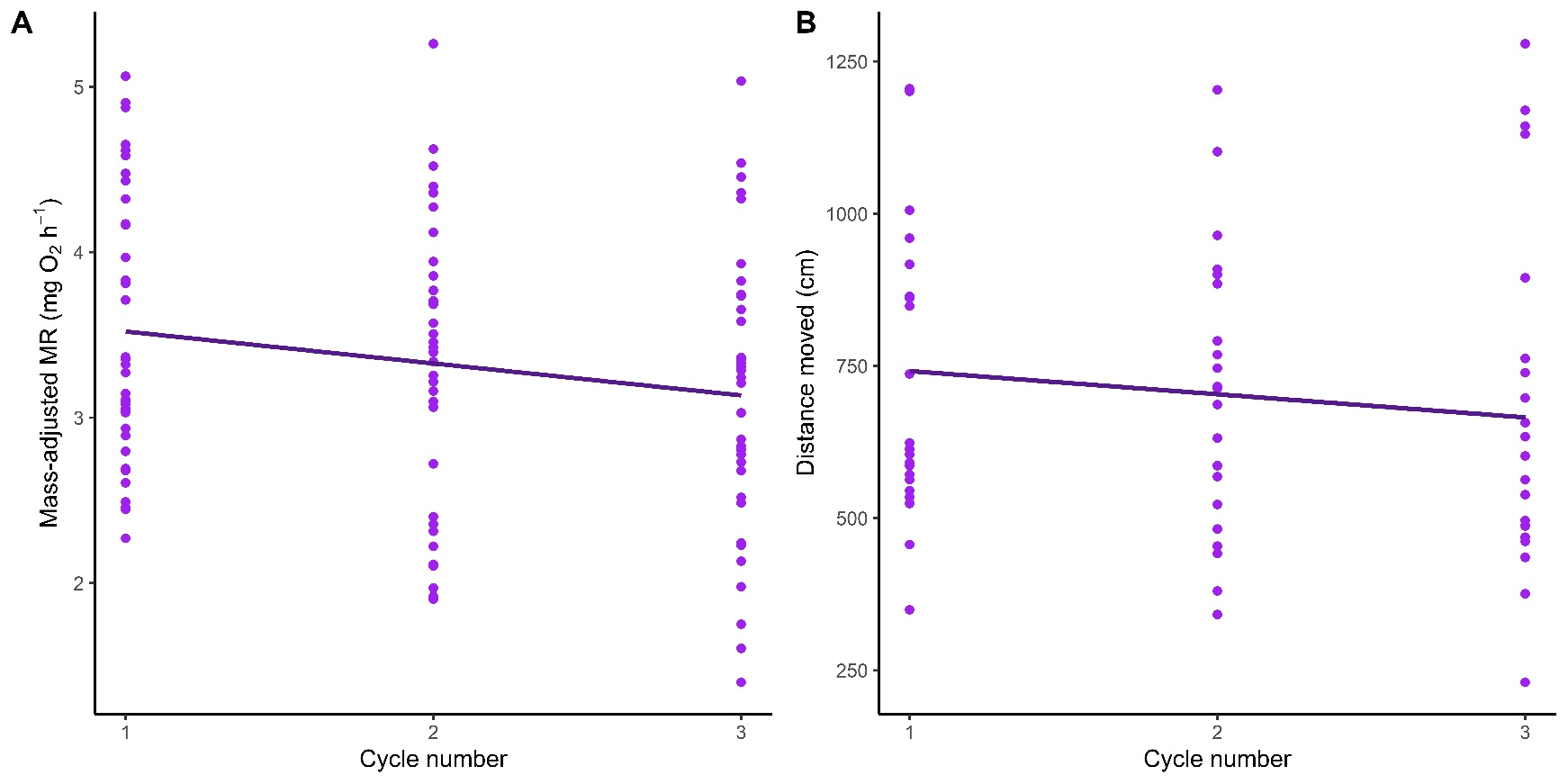
**Supplementary Fig. 2. Effect of repeated exposure to virtual stimuli on A. mass-adjusted metabolic rate (MR) and B. distance moved.**

**
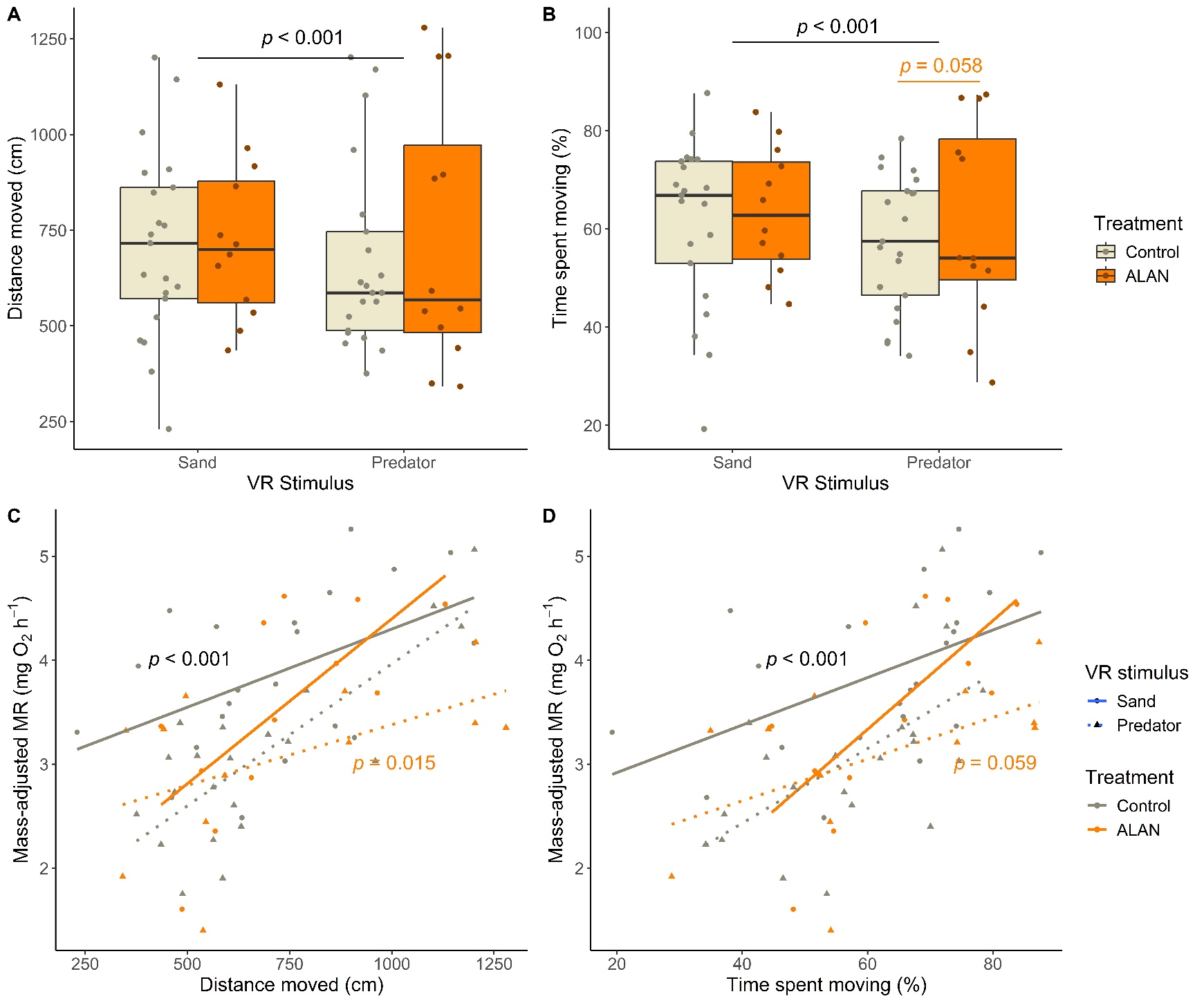
Supplementary Fig. 3. Effect of artificial light at night and virtual reality (VR) predation stimulus on *Amphiprion maohiensis* A. Distance moved, B. Percentage time spent moving, C. Relationship between mass-adjusted metabolic rate and distance moved, and D. Relationship between mass-adjusted metabolic rate (MR) and percentage time spent moving.**
